# Antimicrobial Peptides in Wound Healing and Skin Regeneration: Dual Roles in Immunity and Microbial Defense

**DOI:** 10.3390/ijms26135920

**Published:** 2025-06-20

**Authors:** Siti Balqis Adnan, Manira Maarof, Mh Busra Fauzi, Nur Izzah Md Fadilah

**Affiliations:** 1 Department of Tissue Engineering and Regenerative Medicine (DTERM), Faculty of Medicine, Universiti Kebangsaan Malaysia, Cheras 56000, Kuala Lumpur, Malaysia; balqisadnan179@gmail.com (S.B.A.); manira@ukm.edu.my (M.M.); fauzibusra@ukm.edu.my (M.B.F.); 2 Advance Bioactive Materials-Cells UKM Research Group, Universiti Kebangsaan Malaysia, Bangi 43600, Selangor, Malaysia; 3 Ageing and Degenerative Disease UKM Research Group, Universiti Kebangsaan Malaysia, Bangi 43600, Selangor, Malaysia; 4 Pharmaceuticals and Pharmacy Practice UKM Research Group, Universiti Kebangsaan Malaysia, Bangi 43600, Selangor, Malaysia

**Keywords:** antimicrobial peptides, immune modulation, wound healing, skin regeneration, therapeutic applications

## Abstract

Although penicillin transformed antibiotic therapy, rising antimicrobial resistance (AMR) has limited its effectiveness, creating a need for new approaches in wound healing. Antimicrobial peptides (AMPs) are promising candidates due to their rapid membrane-disrupting action, immunomodulatory effects, and ability to target drug-resistant pathogens, though their specific roles in promoting wound healing are still not fully understood. This review aims to provide a comprehensive synthesis of the current evidence on the dual role of AMPs as both antimicrobial and immunomodulatory agents in the context of wound healing. Recent studies published between 2020 and 2025 were comprehensively reviewed, focusing on the mechanisms by which AMPs contribute to pathogen elimination, immune regulation, tissue repair, and inflammation resolution. AMPs not only exhibit rapid membrane-disruptive activities against a wide range of pathogens but also influence immune cell behavior, particularly by promoting macrophage polarization toward a reparative M2 phenotype, modulating cytokine and chemokine network, and maintaining T-cell homeostasis. Their ability to simultaneously control infection and regulate inflammation positions AMPs as promising candidates for advanced wound care strategies. The dual antimicrobial and immunomodulatory functions of AMPs represent a synergistic mechanism essential for effective wound recovery. Understanding and harnessing these properties can drive the development of innovative therapies, such as AMP-integrated smart biomaterials and targeted peptide delivery systems, offering new solutions for both acute and chronic wound management.

## 1. Introduction

Often referred to as the “silent epidemic”, chronic wounds pose a persistent challenge, necessitating pragmatic research strategies to develop effective translational medical solutions. Despite advances in wound healing, chronic wounds remain a major global healthcare burden, straining systems with their high prevalence and costs [1]. In 2019, approximately 10.5 million U.S. Medicare beneficiaries were affected by chronic non-healing wounds, reflecting an increase of 2.3 million compared to 2014. This rise was particularly notable in the prevalence of pressure ulcers (PUs), chronic ulcers, diabetic foot ulcers (DFUs), and surgical infections, with a significant increase observed among younger beneficiaries (<65 years) [2]. Chronic wounds are defined as wounds that fail to progress through the orderly and timely reparative healing process within the expected timeframe, typically persisting beyond 4 to 12 weeks. Unlike acute wounds, which follow a well-regulated sequence of hemostasis, inflammation, proliferation, and remodeling, chronic wounds remain in a prolonged inflammatory state, leading to impaired tissue repair and persistent tissue damage [3]. The pathogenesis of chronic wounds is multifactorial, often influenced by underlying systemic conditions such as diabetes mellitus, vascular insufficiency, prolonged pressure, and infection, which disrupt normal wound healing mechanisms. Clinically, chronic wounds encompass a broad spectrum of conditions, including DFUs, venous leg ulcers (VLUs), and pressure ulcers [4,5,6,7]. Given their significant impact on patient morbidity and healthcare costs, chronic wounds remain a critical challenge in clinical practice and wound management research.

Chronic wounds are highly susceptible to infection, significantly impairing healing and contributing to prolonged wound persistence [8]. The compromised skin barrier in chronic wounds provides an entry point for pathogenic microorganisms, such as bacteria (e.g., *Staphylococcus aureus*, *Pseudomonas aeruginosa*, and *Streptococcus pyogenes*), fungi (mainly *Candida* species), and certain viruses, leading to the formation of biofilms, which are structured microbial communities that exhibit heightened resistance to host immune responses and antimicrobial treatments. Persistent infection sustains a prolonged inflammatory state, preventing the transition to the proliferative and remodeling phases of healing. Antibiotics have long been regarded as the gold standard for treating microbial infections, including those affecting skin wounds, due to their ability to eliminate pathogens and control infection-related inflammation. In chronic wounds, systemic and topical antibiotics are commonly used to target Gram-positive and Gram-negative bacteria. However, their efficacy is increasingly compromised by antimicrobial resistance (AMR), a growing global health threat driven by microbial genetic adaptations and selective pressures such as the overuse and misuse of antibiotics. Resistant pathogens, including methicillin-resistant *Staphylococcus aureus* (MRSA), multidrug-resistant *Pseudomonas aeruginosa*, and vancomycin-resistant *Enterococcus* (VRE), form biofilms within wounds, reducing antibiotic penetration and efficacy. Consequently, infections persist, sustaining a prolonged inflammatory state that disrupts the normal wound-healing cascade and impairs tissue regeneration [5,7,9,10,11].

**Figure 1:**
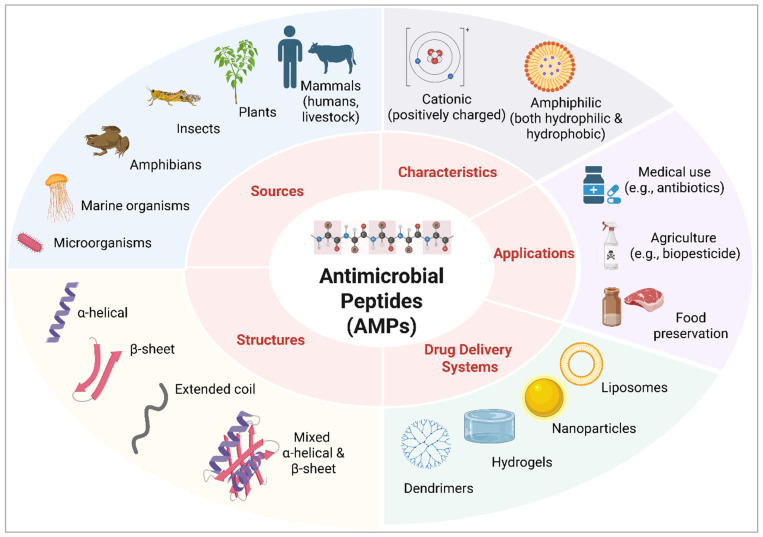
Overview of AMPs. Sources of AMPs and their examples: AMPs can be naturally isolated from various sources (humans: cathelicidins and histatins; livestock such as cows: tritrpticin; plants: thionins and defensins; insects: cecropins; amphibians: magainins —found in the skin of the African clawed frog, *Xenopus laevis*; marine organisms: aurelin from mesoglea of the scyphoid jellyfish *Aurelia aurita*; and microorganisms: bacteriocins from *Lactococcus lactis* subspecies). Structural features: AMPs can adopt different structures: α-helical (e.g., LL-37), β-sheet (e.g., human α-defensin 6), loop/extended coil (e.g., thanatin/indolicidin), or hybrid structures of α-helical and β-sheet conformations (e.g., human β-defensin-2). Key physicochemical characteristics: AMPs are cationic and amphiphilic. General applications: medical use, agriculture, and food preservation. Current strategies for drug delivery: dendrimers, hydrogels, nanoparticles, and liposomes.

Given the limitations of conventional antibiotics and the escalating threat of antimicrobial resistance, there is an urgent need for versatile alternative therapies that effectively combat resistant microbial strains. Antimicrobial peptides (AMPs), a class of small, naturally occurring peptides, have emerged as promising candidates due to their broad-spectrum antimicrobial activity, ability to target antibiotic-resistant pathogens, and multifunctional roles in immune modulation, wound healing, and tissue regeneration. The lower propensity of AMPs to acquire microbial resistance is primarily due to preferential attack on the microbial membrane in addition to their multiple modes of action against microorganisms, which allow them to bypass common resistance mechanisms [12,13,14]. AMPs are typically amphipathic, with cationic residues forming the hydrophilic region and hydrophobic residues occupying the opposite face. This structural polarity, combined with their net positive charge, enables them to interact with and disrupt negatively charged bacterial membranes (Figure 1) [15,16]. This has led to an interest in exploring AMPs as a potentially safer solution that could produce similar or superior effects to antibiotics, albeit with minimal risks of antimicrobial resistance. Unlike traditional antibiotics, AMPs disrupt microbial membranes and interfere with intracellular processes, offering a multifaceted attack on pathogens [17]. Additionally, they modulate the host immune response, making them a compelling alternative to overcome AMR-associated challenges while promoting efficient wound healing and infection control [13,18].

Figure 1 illustrates an overview of AMPs, including their sources, structures, characteristics, general applications, and drug delivery systems. AMPs are widely distributed across life forms and serve as essential components of innate immunity. In humans and other mammals, AMPs such as defensins, cathelicidins (e.g., LL-37), histatins, and tritrpticin are produced by immune and epithelial cells [19,20,21,22]. Invertebrates like insects and crustaceans generate AMPs such as cecropins (purified from fat body cells or hemocytes in *Drosophila*, bees, and guppy silkworms) [19,23] and tachyplesins (isolated from horseshoe crab hemocytes) [24], respectively, while plants produce defensins, thionins, and snakins from their leaves, stems, and seeds [19]. Amphibians, especially frogs and toads, secrete a diverse range of AMPs, such as magainins, from their skin [19]. Microorganisms, including bacteria, release bacteriocins (produced by *Lactococcus lactis*) [19], and marine species such as scyphoid jellyfish *Aurelia aurita* generate aurelin [25]. Further, synthetic peptides, such as pexiganan, an analog of the magainin peptides, are also being developed for therapeutic applications [26]. Based on structures, the main categories of AMPs include α-helical peptides (e.g., LL-37), β-sheet peptides (e.g., human α-defensin 6) [27], loop or extended conformations (e.g., thanatin and indolicidin, respectively) [28], or peptides with both α-helical and β-sheet conformations (e.g., human β-defensin-2). These structural variations enable AMPs to interact with microbial membranes, leading to cell lysis or immune modulation [27,29].

Furthermore, Figure 1 also illustrates the various drug delivery systems (DDSs) into which AMPs can be incorporated. DDSs are formulations or devices designed to deliver therapeutic agents to targeted sites within the body [30]. Recent research has focused on advanced DDSs such as dendrimers, hydrogels, nanoparticles, and liposomes, which can encapsulate AMPs either alone or alongside other therapeutic agents. These systems aim to enhance the stability, bioavailability, and targeted delivery of AMPs, improving their therapeutic potential [31,32,33]. AMPs have been extensively studied for their diverse applications across multiple sectors. In the pharmaceutical field, AMPs are being developed as alternatives or adjuncts to traditional antibiotics [15], offering potential treatments for antibiotic-resistant infections and chronic wounds. In agriculture, AMPs serve as biopesticides to protect crops from bacterial, fungal, and viral pathogens, reducing the reliance on chemical pesticides and promoting sustainable farming practices [34]. In the food industry, AMPs are utilized for food preservation due to their ability to inhibit spoilage microorganisms and extend shelf life, offering a natural and safe alternative to synthetic preservatives [35]. These broad applications underscore the versatility and growing importance of AMPs in addressing global challenges related to health, food security, and environmental sustainability.

**Figure 2:**
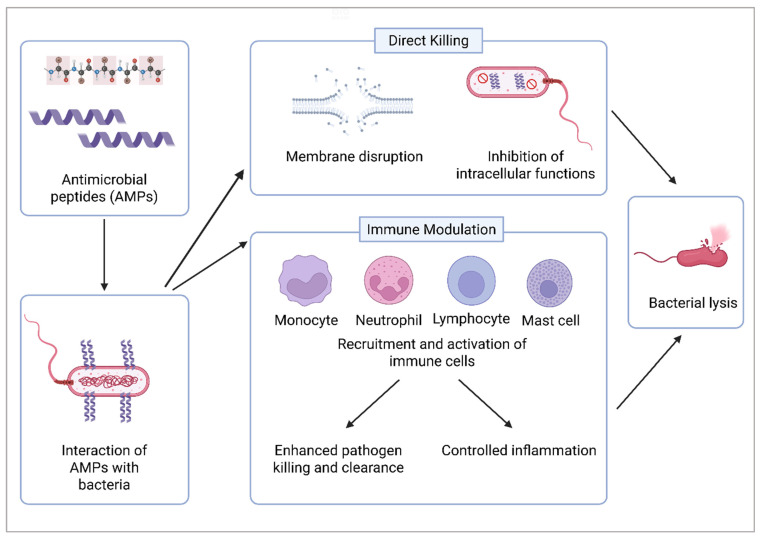
Overview of the mechanisms of AMPs in eliminating pathogens: AMPs eliminate pathogens primarily through two key mechanisms: direct microbial killing and immune system modulation. Their amphipathic structures allow them to interact with and disrupt microbial membranes, leading to cell lysis through membrane destabilization and inhibition of intracellular functions. In addition to this direct action, AMPs also modulate the host immune response, which enhances pathogen killing and clearance and balances inflammation. Together, these dual functions enable AMPs to efficiently combat infections while regulating the immune system.

In addition, Figure 2 provides a detailed overview of the mechanisms by which AMPs target and eliminate pathogens. These mechanisms include direct bactericidal actions, such as disrupting microbial membranes and interfering with intracellular processes, as well as immunomodulatory effects that enhance the host’s immune response, promoting pathogen clearance and tissue repair. This highlights AMPs as promising candidates for dual-function therapeutic applications. The development of innovative therapeutic approaches that not only target pathogens but also modulate the immune response to facilitate effective wound healing is imperative. The potential of AMPs as an alternative to conventional antibiotics has been extensively investigated [12,13,14,15,16,18]. Beyond their well-established antibacterial activity, AMPs also modulate immune responses, underscoring their multifunctional role in host defense. This includes acting as signaling molecules that recruit immune cells, promoting wound healing, and regulating pro- and anti-inflammatory cytokine production. Such functions position AMPs not only as natural antibiotics but also as key mediators of immune homeostasis and tissue repair. While Section 3 and Section 4 will further explore these roles, it is important to note that the immunomodulatory functions of AMPs in wound healing and skin regeneration remain incompletely understood. By influencing processes such as neutrophil recruitment, macrophage polarization, and cytokine regulation, AMPs orchestrate critical stages of tissue regeneration [36,37].

By stimulating angiogenesis, promoting keratinocyte migration, and enhancing fibroblast activity, which are key processes for wound closure and skin restoration, AMPs can directly contribute to tissue remodeling alongside their immunomodulatory roles [38,39,40,41,42,43]. This review examines how AMPs coordinate the transition from inflammation to proliferation during wound healing, emphasizing the molecular interplay between immune regulation and tissue regeneration. Unlike previous studies that often treat these roles separately, we explore how the antimicrobial and immunomodulatory functions of AMPs intersect to drive effective wound resolution. By highlighting this dual functionality, we underscore their therapeutic potential not only as alternatives to conventional antibiotics but also as modulators of the wound microenvironment, offering new directions for treating both acute and chronic wounds.

## 2. Data Extraction Management

A literature search was conducted within six years of publications (2020–2025) through PubMed, Web of Science (WoS), and Scopus. The search strategy used the terms ‘antimicrobial peptides’, ‘immune modulation’, ‘wound healing’, ‘skin regeneration’ and ‘therapeutic applications’. The exclusion criteria for this review were all secondary literature and any original articles written and submitted in languages other than English.

## 3. Complex Interplay of Immune Responses and Wound Healing

### 3.1. Overview of Immune Response in Acute Wound Healing

The invasion of pathogens in wounds triggers a complex cascade of immune responses, highlighting the intricate interplay between microbial infection mechanisms, immune activation, and the wound-healing process. The immune system plays a central role in wound healing, orchestrating a finely tuned balance between inflammation, tissue repair, and infection control. In response to tissue injury, acute wounds undergo immediate vasoconstriction to minimize blood loss and prevent exsanguination [38,39]. Platelets, the key mediators of hemostasis and coagulation, rapidly aggregate at the injury site, initiating the hemostasis phase of wound healing. This aggregation leads to the formation of a fibrin clot, which serves as a barrier against further bleeding and microbial invasion [38,40,41]. The degranulation of platelets triggers the release of factors such as platelet factor 4 (PF4), platelet-derived growth factor (PDGF), vascular endothelial growth factor (VEGF), and transforming growth factor-β1 (TGF-β1) [42]. Additionally, the activated platelets also secrete pro-inflammatory cytokines, including interleukin-1 alpha (IL-1α), interleukin-1 beta (IL-1β), interleukin-6 (IL-6), C-X-C motif chemokine ligand 8 (CXCL8), and tumor necrosis factor-alpha (TNF-α). These molecules play a crucial role in recruiting immune cells to the wound milieu, thereby facilitating the subsequent inflammatory phase of healing [6,43,44,45,46,47,48].

The acute inflammatory response following injury is triggered by damage-associated molecular patterns (DAMPs), which are released by apoptotic or necrotic cells [49]. These DAMPs activate pathogen recognition receptors, such as toll-like receptors (TLRs), which are expressed on various immune and structural cells, including neutrophils, resident monocytes/macrophages, dendritic cells, mast cells, T cells, and keratinocytes. The recognition of DAMPs by TLRs initiates intracellular signaling cascades, leading to the production of cytokines and other immune mediators that facilitate pathogen elimination and promote the activation of adaptive immune responses [44,50]. During the inflammatory phase, neutrophils, known as the immune system’s first responders, rapidly migrate to the injury site to combat pathogens and initiate the inflammatory response. They release reactive oxygen species (ROS) and AMPs to neutralize invading pathogens while simultaneously secreting pro-inflammatory cytokines and chemokines, including IL-1β, IL-6, CXCL2, CXCL8, TNF-α, and VEGF-A [48]. In addition to maintaining immune activation and increasing inflammation, these signaling molecules are also vital for fibroblast and keratinocyte proliferation and angiogenesis, all of which are necessary for efficient wound healing [6,43,44,45,46].

Macrophages play a pivotal role in acute wound healing, orchestrating the transition from the inflammatory phase to the tissue repair phase. These highly plastic immune cells exhibit functional heterogeneity, dynamically shifting between pro-inflammatory (M1) and pro-healing (M2) phenotypes in response to the wound microenvironment. Initially, circulating monocytes recruited to the inflamed tissue can be classified into classical (CD14^+^CD16^−^) pro-inflammatory monocytes and anti-inflammatory (CD14^low/−^CD16^+^) monocytes. The classical monocytes predominantly differentiate into M1 macrophages, which amplify the inflammatory response, whereas the anti-inflammatory monocytes primarily give rise to M2 macrophages, which are involved in tissue repair and the resolution of inflammation [51]. The M1 phenotype produces inflammatory mediators, including nitric oxide (NO), ROS, IL-1, IL-6, TNF-α, and matrix metalloproteinases (MMP-2 and MMP-9), which contribute to the inflammatory response, degradation of pathogens, and necrotic debris clearance [6,43,44,45].

The proliferation phase is characterized by the transition from inflammation to healing, re-epithelialization, angiogenesis, and granulation tissue formation [52,53,54]. The immune system plays a central role in this phase by coordinating fibroblast activation, keratinocyte proliferation, angiogenesis, and extracellular matrix (ECM) remodeling [38,55,56]. As the healing progresses, the polarization of M1 into M2 phenotype occurs, shifting the balance to the anti-inflammatory state. The pro-wound healing macrophages, alternatively known as activated macrophages, promote tissue repair and neovascularization by releasing factors such as PDGF, TGF-β, insulin-like growth factor 1 (IGF-1), and VEGF. The release of TGF-β and PDGF promotes fibroblast activities, including migration, proliferation, and ECM production, such as collagen, ultimately strengthening the tissue structure. Additionally, wound-associated macrophages facilitate the restoration of the epidermal barrier through the secretion of IL-10 and PDGF-β, which stimulate keratinocyte activity and accelerate re-epithelialization [57,58].

Proper immune regulation during this phase ensures efficient wound closure while preventing chronic wounds or excessive scarring. As the wound recovery progresses, the immune response gradually diminishes through the apoptosis of excess immune cells, marking the transition to the remodeling phase of wound recovery. During this phase, the wound undergoes structural reinforcement and maturation through collagen remodeling and the resolution of angiogenesis, while fibroblast apoptosis helps limit excessive scar formation. Additionally, matrix metalloproteinases (MMPs) and other tissue-remodeling enzymes degrade ECM components to support proper tissue restructuring and functional restoration. Ultimately, the immune system plays a critical and dynamic role in wound healing, orchestrating each process phase. In summary, the immune system works together to eliminate pathogens, clear cellular debris, and regulate angiogenesis, fibroblast activation, and ECM remodeling, ensuring effective tissue repair [6,43,44,45].

### 3.2. Overview of Immune Response in Chronic Wound Healing

The wound healing process consists of four distinct phases: hemostasis, inflammation, proliferation, and maturation, all of which must be precisely coordinated to achieve proper tissue repair [59]. Chronic wounds, which include conditions such as VLUs, DFUs, and pressure ulcers [4,5,6,7], are characterized by the failure to advance through the typical phases of healing. This results in persistent pathological inflammation, continuous infection, and necrosis, ultimately impairing subsequent phases of tissue repair. Various local and systemic factors, including infections, impaired blood flow, underlying diseases such as diabetes, and compromised immune functions, can disrupt the normal wound-healing process, contributing to chronic wound formation [44,60]. In acute wounds, inflammation is an essential phase of the healing process, during which the immune system is activated to clear debris and pathogens and initiate tissue repair. However, infections marked by bacterial biofilms can intensify and prolong this inflammatory response as the immune system attempts to eradicate the persistent pathogens, further hindering the healing process. The microenvironment of chronic wounds is characterized by an abundance of pro-inflammatory macrophages, excessive production of inflammatory mediators (e.g., TNF-α and IL-1β), heightened MMP activity, increased levels of ROS, and disruptions in proteolytic balance, ultimately causing tissue damage and impairing keratinocyte proliferation and tissue re-epithelialization [44,61,62,63,64].

Immune dysregulation during the healing process can result in prolonged inflammation and impaired tissue regeneration, ultimately contributing to the delay in healing [44,61,65,66,67]. Given that inflammation is a hallmark of chronic wounds and is intricately connected to immune regulation, excessive neutrophil infiltration acts as a biomarker of non-healing wounds, highlighting the complex dynamics of the immune response. In a healthy wound, neutrophils help remove debris and prevent infection; however, their activity is tightly regulated and they are often cleared from the wound as healing progresses. Abundant neutrophils in chronic wounds contribute to a persistent inflammatory state, hindering healing by releasing destructive enzymes (e.g., proteases such as elastases) and ROS, while also forming neutrophil extracellular traps (NETs), which may damage surrounding tissues and impair angiogenesis [61,68,69,70]. Macrophages, which are crucial regulators of wound healing, play a key role in inflammation and tissue repair. In chronic wounds, the transition from the pro-inflammatory state phenotype (M1 macrophages) to the anti-inflammatory state phenotype (M2 macrophages) is often impaired, stalling the inflammatory phase. Consequently, an abundance of M1 macrophages leads to high levels of pro-inflammatory cytokines and destruction of the ECM scaffold, which further contribute to the persistent inflammation and delayed tissue repair, respectively [71,72,73].

In chronic wounds, the tightly regulated balance of cytokines and chemokines becomes severely disrupted, contributing to impaired healing. Under normal conditions, these signaling molecules coordinate the timely recruitment and activation of immune cells, promoting progression through the phases of wound repair [44,61]. However, in chronic wounds, prolonged and excessive production of pro-inflammatory cytokines such as TNF-α, IL-1β, and IL-6 sustains a persistent inflammatory environment. Concurrently, elevated levels of chemokines such as CXCL8 (IL-8) drive continuous neutrophil recruitment, further amplifying tissue damage through the release of proteases and ROS [44,61,74,75,76]. This pro-inflammatory dominance is accompanied by insufficient production of anti-inflammatory cytokines such as IL-10 and TGF-β, which are crucial for resolving inflammation and initiating tissue regeneration [77,78,79,80]. The resulting imbalance not only perpetuates immune cell infiltration and local inflammation but also interferes with processes like keratinocyte proliferation, fibroblast function, and ECM remodeling. Collectively, the dysregulation of cytokine and chemokine networks creates a hostile wound microenvironment that impedes normal healing and contributes to the chronicity of non-healing wounds [44,61].

**Figure 3:**
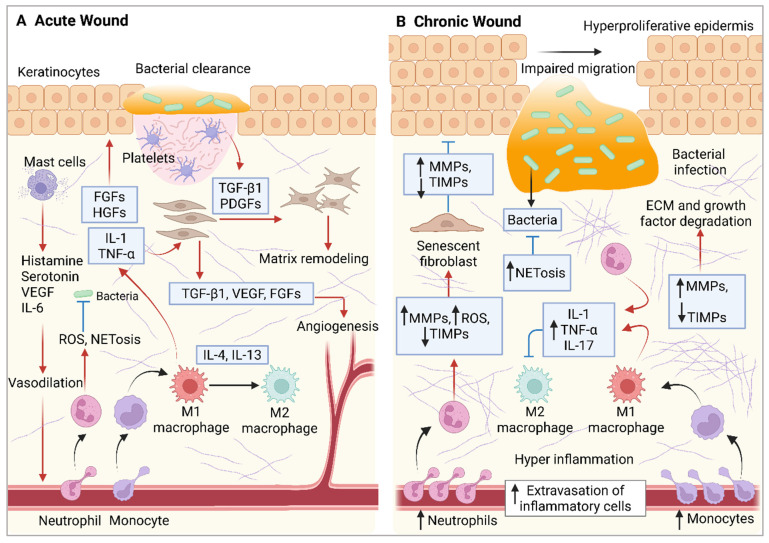
Summary of immune responses in acute versus chronic wound healing. (**A**) Acute wound healing involves a tightly regulated sequence of overlapping phases: hemostasis, inflammation, proliferation/ECM deposition, and tissue remodeling. Neutrophils and macrophages are central to this process, while T cells and platelets also contribute significantly. (**B**) In chronic wounds, elevated levels of inflammatory cells and the presence of biofilms hinder the re-establishment of tissue homeostasis. The excessive release of inflammatory mediators leads to the degradation of growth factors and ECM and impairs the transition of macrophages to a pro-healing phenotype. This creates a self-perpetuating cycle that obstructs proper wound resolution. Black arrows represent differentiation, blue arrows indicate inhibition, and red arrows show induction.

Therefore, advancing immunomodulatory approaches targeting the immune response, such as regulating neutrophil activity, enhancing macrophage polarization, balancing cytokine and chemokine responses, and ensuring proper T cell function, is essential for promoting effective wound healing. Figure 3 summarizes the immune responses involved in acute and chronic wound healing. The persistent inflammatory state and biofilm formation create significant barriers to effective wound resolution, making chronic wounds particularly challenging to treat. Despite the integral role of the immune system in eradicating pathogens from the wound site, the chronicity of persistent wounds can be driven by a combination of immune dysfunction with other contributing factors such as bacterial colonization. Therefore, targeting immune modulation alongside infection control is crucial for enhancing wound healing outcomes and preventing chronic wound complications [44]. Recent studies have reported the immunomodulatory role of AMPs, offering promising avenues to combat infections, regulate immune responses, and accelerate wound closure [81].

## 4. Dual Role of Antimicrobial Peptides in Wound Healing and Skin Regeneration

**Figure 4:**
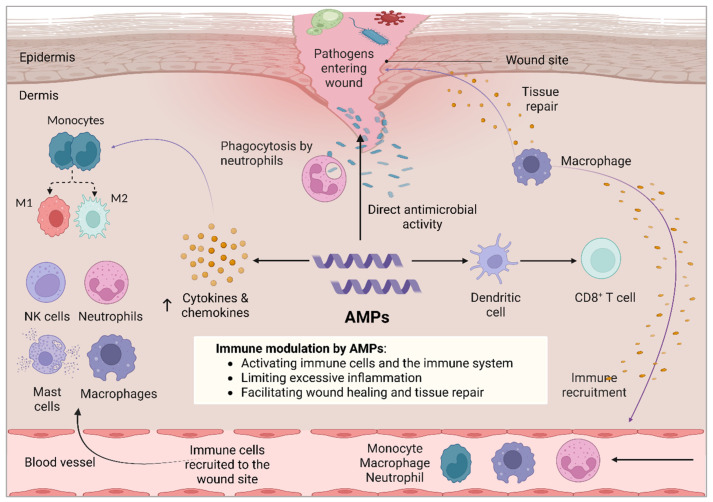
AMPs play a dual role in host defense by combining direct antimicrobial action with immune system modulation. Their primary antimicrobial mechanism involves disrupting the integrity of microbial membranes, leading to rapid pathogen lysis and death. Concurrently, AMPs enhance the host immune response by modulating both innate and adaptive immunity. Upon infection or injury, AMPs help target pathogens at the wound site, not only by directly attacking microbial invaders but also by facilitating their clearance through neutrophil-mediated phagocytosis. Additionally, AMPs stimulate innate immune components by recruiting and activating immune cells such as macrophages, mast cells, neutrophils, and natural killer (NK) cells. These activated cells secrete pro-inflammatory cytokines and chemokines, amplifying the immune response and promoting the efficient removal of pathogens. Beyond innate immunity, AMPs also bridge the transition to adaptive immune responses by activating dendritic cells, which process and present antigens to naïve T cells. This interaction stimulates the proliferation of cytotoxic CD8^+^ T cells, enhancing the targeted elimination of infected or abnormal host cells. Through this multifaceted role, AMPs not only eliminate pathogens but also orchestrate a coordinated immune response, contributing to tissue protection and repair. Purple arrows denote the action of cytokines and chemokines on immune cell recruitment, activation, and microbial clearance. Black arrows indicate the functional roles of AMPs, including direct antimicrobial activity, immune modulation, cytokine induction, and activation of immune cells. Orange dots represent cytokines and chemokines secreted primarily by macrophages that mediate inflammation and immune cell recruitment. Note: ”Immune recruitment” in the figure specifically refers to the recruitment of immune cells to the site of infection or injury.

As bacterial infections impede wound healing and antibiotic resistance continue to escalate, AMPs have emerged as a promising alternative therapy. Understanding the distinct stages of wound healing is essential for optimizing AMP-based treatments, which potentiate their dual ability [82]. AMPs exhibit direct antimicrobial properties and play a crucial role in modulating the immune system, making them valuable in wound healing and infection control. Their immunomodulatory functions include regulating cytokine production, influencing macrophage polarization, and enhancing immune cell recruitment [44,45]. Table 1 presents examples of AMPs with reported immunomodulatory activities, highlighting their diverse roles in balancing inflammation and promoting tissue repair. Figure 4 summarizes the mechanisms by which AMPs modulate immunity to combat infections and promote wound healing and tissue repair. AMPs modulate immunity through multiple mechanisms that both combat infections and promote wound healing. They enhance innate immunity by recruiting and activating immune cells such as macrophages, neutrophils, mast cells, and natural killer (NK) cells, leading to the release of cytokines and chemokines that drive inflammation and pathogen clearance. AMPs also regulate cytokine production and promote macrophage polarization from a pro-inflammatory (M1) to an anti-inflammatory (M2) phenotype, which supports inflammation resolution and tissue regeneration. Additionally, AMPs influence adaptive immunity by activating dendritic cells, which present antigens to T cells and stimulate cytotoxic CD8^+^ T cell responses. Together, these actions help eliminate pathogens, control inflammation, and accelerate tissue repair.

### 4.1. AMPs Modulate Macrophage Polarization in Wound Healing

In chronic wounds such as diabetic ulcers, the transition of macrophages from a pro-inflammatory (M1) to an anti-inflammatory (M2) phenotype is often impaired. This disrupts inflammation resolution, increasing susceptibility to microbial infections and biofilm formation, ultimately delaying tissue repair. Peptide-based therapeutics, particularly AMPs, have shown promise in modulating immune responses by promoting macrophage polarization while simultaneously exerting antimicrobial effects [44,45]. CSP32, a novel oligomeric antimicrobial peptide derived from bacitracin, which is a macrocyclic peptide antibiotic produced by *Bacillus* species, was investigated by Ji et al. [97] for its immunomodulatory effects, particularly in macrophage polarization. While bacitracin is traditionally used as a topical antibiotic effective against Gram-positive bacteria, recent studies have explored its enhanced antibacterial properties, with rationally designed variants demonstrating potent activity against VRE [98]. In this study, CSP32 was shown to direct the polarization of murine macrophage-like RAW 264.7 cells toward the pro-inflammatory M1 phenotype. This effect was accompanied by a significant upregulation of genes associated with macrophage polarization, including interleukins, CC chemokine ligands (CCLs), cluster of differentiation markers, and other immune regulatory genes; each showing over a 1.5-fold increase compared to control cells. These findings were further supported by microarray analysis and ELISA, which confirmed increased expression and secretion of M1 macrophage markers such as NO, TNF-α, and IL-1β. Both mRNA and protein levels of these markers were significantly elevated upon CSP32 treatment, in alignment with ELISA data [97]. Collectively, the study underscores the dual role of CSP32 as both an antimicrobial and an immunomodulatory agent, highlighting its potential to enhance the early inflammatory response critical for efficient wound healing and subsequent tissue repair.

**Figure 5:**
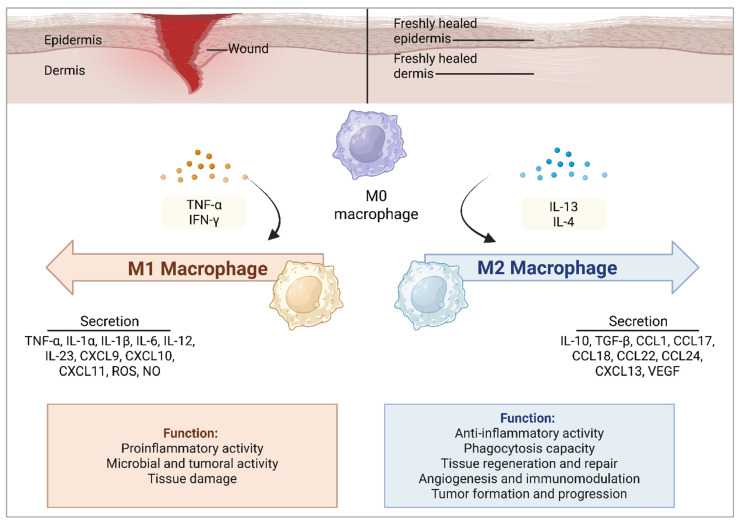
Overview of mechanisms of macrophage polarization: Macrophages shift between pro-inflammatory (M1) and anti-inflammatory (M2) states in response to signals from their environment, such as cytokines. This dynamic change regulates infection control, inflammation, and tissue repair. During the early stages of wound healing, M1 macrophages are typically induced by IFN-γ and TNF-α, leading to a pro-inflammatory phenotype. These cells secrete high levels of pro-inflammatory cytokines (e.g., IL-1β, IL-6, and TNF-α), ROS, and nitric oxide, which are crucial for proinflammatory activity. As the inflammatory phase resolves, M1 macrophages undergo a phenotypic switch to the M2 subtype, a transition essential for key physiological processes such as angiogenesis and tissue regeneration that support effective wound healing. M2 macrophages are induced by anti-inflammatory cytokines, including IL-13 and IL-14. Upon activation, they secrete regulatory cytokines such as IL-10 and TGF-β, which facilitate tissue repair, promote angiogenesis, and enhance collagen deposition to restore tissue integrity.

Similarly, nisin, a naturally occurring AMP produced by *L. lactis*, exhibits potent inhibitory activity against both Gram-positive and Gram-negative bacteria, notably *S. aureus* and *E. coli*, respectively. It has been widely explored in combination therapies with conventional antibiotics or biomaterials such as polymers, often resulting in additive or synergistic antimicrobial effects. For instance, studies combining nisin with the antimicrobial peptide P10 and traditional antibiotics have demonstrated enhanced efficacy against extensively drug-resistant *Acinetobacter baumannii* and colistin-resistant *Pseudomonas aeruginosa* isolates, suggesting synergistic interactions that improve antimicrobial outcomes [99]. Beyond its antimicrobial properties, nisin also contributes to immune modulation, particularly by influencing macrophage polarization. Yang and colleagues demonstrated that NPF@MN, a novel multifunctional hyaluronic acid microneedle patch loaded with nisin, exhibited potent antibacterial and anti-biofilm activities in both in vitro and in vivo models. Notably, in addition to its antimicrobial effects, the NPF@MN patch also showed significant antioxidant and anti-inflammatory properties through ROS scavenging. This activity contributed to the modulation of macrophage polarization from the pro-inflammatory M1 phenotype to the pro-repair M2 phenotype, thereby enhancing epithelial regeneration and collagen deposition. Notably, this microneedle system not only inhibited bacterial infection but also promoted M2 macrophage polarization, accelerating wound healing by modulating the immune microenvironment and influencing the insulin signaling pathway in biofilm-infected diabetic wounds [100]. These findings provide a valuable foundation for further research into the integration of nisin with biomaterials, such that nisin is not only effective in eliciting antimicrobial effects but also capable of modulating the immune response, particularly by supporting macrophage transition and balancing inflammatory responses. These two aspects are essential for wound healing and tissue regeneration [87]. Figure 5 illustrates the process of macrophage polarization, highlighting the shift from M1 to M2 states in response to microenvironmental signals.

### 4.2. AMPs Modulate Cytokine and Chemokine Regulation in Wound Healing

Cytokines and chemokines regulate wound healing by controlling inflammation, immune cell recruitment, and tissue repair. Pro-inflammatory cytokines (e.g., TNF-α, IL-1β, and IL-6) and chemokines (e.g., CCL2 and CXCL8) initiate immune responses, while anti-inflammatory cytokines (e.g., IL-10 and TGF-β) promote macrophage polarization and tissue regeneration. Additionally, growth factors such as fibroblast growth factors (FGFs) and VEGFs enhance fibroblast activity and angiogenesis, aiding wound closure. In the remodeling phase, cytokines regulate ECM deposition and collagen remodeling. Maintaining a balance between pro- and anti-inflammatory signals is crucial, as dysregulation can result in chronic wounds or excessive fibrosis [44,45,48]. LL-37, also called cathelicidin, is a cationic AMP derived from the cleavage of human cationic antimicrobial peptide-18 (hCAP18). LL-37 has been gaining attention due to its dual role as an antimicrobial agent and immune modulation therapy. Yang et al. [89] investigated the immunoregulatory effects of LL-37/CS—a combination of the antimicrobial peptide LL-37 and a chitosan-based hydrogel—in an in vivo mouse model of deep tissue pressure injury. The injuries were induced using a magnet-based compression system applied under a 12 h on/12 h off schedule over a period of 21 days. The results showed that the LL-37 and LL-37/CS groups exhibited upregulated expression of VEGF-A and TGF-β compared to the CS-only and control groups, ultimately accelerating the healing of deep tissue injuries while also exerting moderate antibacterial activity. Furthermore, the study observed a significant downregulation in the IL-6 and TNF-α expression at the mRNA level on day 14 in the LL-37/CS and LL-37 groups, compared to the control group (*p* < 0.001). These results imply that the upregulation of pro-angiogenic growth factor expression (TGF-β and VEGF-A) potentially enhances blood supply, stimulates granulation tissue formation, and shields against tissue matrix degradation. Meanwhile, LL-37 protects against inflammatory damage by preventing the activation of specific enzymes and key regulators involved in inflammatory responses, resolving chronic inflammation that often hampers the wound-healing process [89].

Other than cathelicidins, other AMPs have also been reported to possess immunomodulatory properties, leading to accelerated wound healing. Silva et al. [91] modified mastoparan-L, the bioactive toxin from the venom of the wasp *Vespula lewisii*, into synthetic AMPs. Beyond its enhanced antibacterial properties, the resulting peptide, Mast-MO, demonstrated significant immunomodulatory effects through multiple mechanisms. Notably, it suppressed pro-inflammatory factors such as TNF-α and IL-6, a crucial function for reducing excessive inflammation and promoting a balanced immune response during the wound healing process. This synthetic derivative of mastoparan-L exhibits AMP-like properties, facilitating increased uptake of antibiotics by compromising bacterial cell membrane integrity [91]. By balancing pro-inflammatory and anti-inflammatory cytokines, AMPs ensure a controlled immune response that prevents chronic inflammation while promoting tissue repair. Additionally, their ability to enhance chemokine signaling facilitates the efficient migration of immune cells, contributing to pathogen clearance and wound resolution. Through these mechanisms, AMPs serve as crucial mediators that bridge innate and adaptive immunity, ultimately accelerating wound healing and improving tissue regeneration. Their immunomodulatory potential highlights AMPs as promising therapeutic candidates for enhancing immune responses and promoting effective wound recovery [44,45]. Cathelicidins, along with other AMPs, hold significant potential as therapeutic agents for chronic wounds by alleviating persistent inflammation and stimulating tissue regeneration in non-healing wounds.

### 4.3. AMPs Modulate Responses by NK Cells in Wound Healing

Natural killer (NK) cells, a subset of innate lymphoid cells, are classically recognized for their role in targeting and eliminating virus-infected or transformed cells via the release of cytotoxic granules containing perforin and granzyme [99]. Emerging evidence highlights their multifaceted involvement in wound healing, encompassing immunoregulatory, antimicrobial, and tissue-supportive functions. The direct role of NK cells in human skin wound healing is unclear; nonetheless, their cytotoxic abilities and immunoregulatory functions allow them to manage infections and inhibit the onset of chronic inflammatory disorders. Within the dynamic milieu of tissue repair, NK cells execute cytotoxic effects and play pivotal roles in modulating inflammation, controlling infection, and promoting tissue regeneration. Upon tissue injury, NK cells are swiftly recruited to the wound site in response to DAMPs and pro-inflammatory cytokines such as IL-12, IL-15, and IL-18, primarily produced by macrophages and dendritic cells. These signals activate NK cells to secrete immunomodulatory cytokines, including interferon-gamma (IFN-γ) and TNF-α [101]. Early production of IFN-γ regulates neutrophil trafficking, influencing both their recruitment to sites of inflammation and their clearance after apoptosis. Additionally, the secreted IFN-γ also enhances macrophage phagocytic capacity, cytotoxic activity, and cytokine secretion, thereby facilitating effective pathogen clearance and regulation of inflammatory responses [101,102,103].

As the healing process advances, rising levels of IL-10 and TGF-β, mainly derived from M2-polarized macrophages, induce a shift in NK cells toward a regulatory, anti-inflammatory phenotype (NKreg), enabling them to contribute to tissue regeneration through the secretion of IL-10. Concurrently, mesenchymal stem cells (MSCs) further fine-tune NK cell function throughout the repair process. In the initial inflammatory phase, MSCs adopt a pro-inflammatory phenotype through activation of TLR3, TLR7, and TLR9, thereby enhancing NK cell cytotoxicity. In later stages, MSCs transition to a regulatory role, promoting the differentiation of pro-inflammatory NK cells into NKreg cells via IDO- and PGE2-dependent pathways or inducing NK cell senescence through IL-6 and TGF-β signaling. These senescent NK cells, in turn, stimulate VEGF production in MSCs, promoting angiogenesis, endothelial proliferation, and overall wound healing [102]. Moreover, the production of lytic granules containing perforin and granzymes by NK cells has been demonstrated to facilitate the clearance of senescent cells accumulating at the injury site, hence expediting healing and preventing fibrosis [103,104].

Together, these interactions underscore the tightly coordinated crosstalk between NK cells and MSCs as a critical component of effective tissue repair. Maintaining a balanced NK cell response is essential for optimal wound healing, as proper NK cell function accelerates tissue regeneration, while their dysfunction or dysregulation can impair wound closure and compromise healing outcomes. Therefore, the development of effective therapeutic agents capable of modulating NK cell activity is crucial for restoring immune homeostasis and promoting optimal tissue repair. In addition to their capacity to activate macrophages and regulate cytokine and chemokine networks, AMPs also modulate the activity of NK cells, which operate synergistically with macrophages to mediate the clearance of infected or damaged host cells. AMPs such as alloferon and its analogues have been shown to modulate NK cell activity by enhancing their cytotoxic functions (via mechanisms such as enhanced secretion of lytic granules) and promoting the production of IFN-γ in both murine and human models. Increased secretion of IFN-γ can indirectly promote wound healing by stimulating the activation of macrophages and other immune cells. Once activated, macrophages play a central role in wound debridement and serve as critical regulators of the healing process [105]. Notably, Rakityanskaya et al. investigated the effects of alloferon on Epstein–Barr Virus (EBV)-infected human saliva samples. Treatment with alloferon resulted in a significant reduction of EBV DNA levels, alongside a marked increase in NK cell-mediated cytotoxicity and a relative rise in NK cell proportions, when compared to samples treated with the antiviral drug valacyclovir (Valtrex) [106]. Thus, by enhancing NK cell-mediated antiviral responses and immune regulation, alloferon may facilitate the resolution of infection-associated inflammation, thereby creating a more favorable environment for effective wound healing.

### 4.4. Other Immunomodulatory Mechanisms of AMPs in Wound Healing

The immune system is a highly intricate and self-regulating defense mechanism composed of a diverse array of immune cells, each playing a crucial role in the distinct phases of wound recovery. AMPs interact with host defense pathways, including TLRs, fine-tuning the immune response to accelerate healing. In a study by Li et al. [92], the short AMP Andersonin-W1 (AW1), which was previously found to exhibit excellent therapeutic effects on acute and diabetic skin wounds in mice, was shown to modulate macrophage inflammation via the TLR4/NF-κB molecular axis by directly binding to TLR4 in the macrophage extracellular region, leading to enhanced re-epithelialization, granulation regeneration, and angiogenesis while demonstrating antibacterial activities. Additionally, the study found the versatile role of AW-1, such that it is capable of modulating macrophage transformative abilities in transitioning from the inflammatory to the proliferative phase, indicating its beneficial role in ensuring a smooth process of wound healing [92]. This study highlights the significant role of AMPs in modulating key signaling pathways involved in wound healing and tissue regeneration, further supporting their dual mechanistic function in both microbial clearance and immune regulation.

A separate study explored the therapeutic potential of an AMP derived from insulin-like growth factor-binding protein 5 (AMP-IBP5) in addressing chronic wounds, particularly in diabetic conditions. The findings revealed that AMP-IBP5 activated mast cells while stimulating keratinocyte and fibroblast proliferation and migration [94], which are essential processes for effective wound healing [107]. Moreover, AMP-IBP5 counteracted the inhibitory effects of high glucose levels on keratinocyte proliferation, migration, and the production of key angiogenic factors, including angiogenin and VEGF. These beneficial effects were attributed to the activation of critical signaling pathways involving the epidermal growth factor receptor (EGFR), signal transducers and activators of transcription (STAT) 1 and 3, and mitogen-activated protein kinases (MAPKs). In vivo studies further demonstrated that AMP-IBP5 significantly accelerated wound closure, upregulated the expression of angiogenic factors, and promoted neovascularization in both normal and diabetic mice, highlighting its potential as a therapeutic agent for chronic wound management [94]. Based on these studies, the role of AMPs in mediating the complex interplay between various signaling pathways and the immune system presents a compelling area of research that warrants further exploration. Understanding how AMPs influence immune cell activation, cytokine regulation, and tissue regeneration through distinct molecular pathways could unlock new therapeutic strategies for enhancing wound healing and combating infections. Continued investigation into these mechanisms may lead to the development of novel AMP-based treatments tailored for immune modulation and tissue repair in chronic and non-healing wounds.

### 4.5. Bridging Innate Immunity and Adaptive Immunity Using AMPs

AMPs play a crucial role in bridging innate and adaptive immunity during wound healing by acting as both direct antimicrobial agents and immune modulators [44,108,109]. During the early stages of wound healing, AMPs such as defensins and cathelicidins enhance innate immunity by disrupting microbial membranes to prevent infection and by promoting the recruitment of immune cells, including neutrophils and macrophages, through chemotactic signaling. These peptides also regulate inflammation by modulating cytokine and chemokine production, ensuring a controlled immune response. As the wound progresses, AMPs influence adaptive immunity by promoting antigen presentation, enhancing dendritic cell maturation, and stimulating T-cell responses. For instance, LL-37 has been shown to activate T cells and modulate B cell function, thereby facilitating long-term immune protection. Additionally, AMPs contribute to tissue regeneration by promoting fibroblast proliferation, angiogenesis, and ECM remodeling. Through their dual function in antimicrobial defense and immune modulation, AMPs serve as key mediators that connect innate and adaptive immunity, ultimately accelerating wound healing and improving tissue repair [110,111].

Minns et al. [90] investigated the role of mouse and human antimicrobial host defense peptide cathelicidin (LL-37/mCRAMP) in promoting the differentiation of Th17 cells in vitro and in vivo. T helper 17 (Th17) cells play a crucial role in wound healing by modulating inflammation, promoting tissue repair, and enhancing host defense. These cells primarily produce IL-17, which helps recruit neutrophils to the wound site, enhancing bacterial clearance and reducing infection risk [112,113]. Additionally, IL-17 stimulates fibroblast activation and ECM deposition, supporting tissue remodeling [114,115]. Th17 cells also regulate angiogenesis by influencing the production of VEGF and other pro-angiogenic factors. However, an imbalance in Th17 cell activity can contribute to excessive inflammation, leading to delayed healing or fibrosis. Therefore, Th17 cells play a dual role in wound healing, balancing immune responses while promoting tissue regeneration. This study provides crucial insights into Th17’s function as an immune modulator in wound healing. As an AMP, cathelicidin not only exhibits direct antimicrobial effects but also actively shapes adaptive immunity by enhancing Th17 cell differentiation through the upregulation of aryl hydrocarbon receptor (AHR) and RORγt in a TGF-β1-dependent manner. This modulation of Th17 responses is significant in wound healing, as IL-17, the signature cytokine of Th17 cells, plays a key role in neutrophil recruitment, fibroblast activation, and ECM deposition, all of which contribute to tissue repair. Additionally, cathelicidin-mediated Th17 differentiation supports angiogenesis by influencing VEGF production, further accelerating wound closure. However, a finely tuned balance is essential, as excessive Th17 activity may lead to chronic inflammation or fibrosis, underscoring the importance of immunomodulatory strategies in wound therapy. Overall, these findings highlight the multifaceted role of cathelicidin in linking innate and adaptive immunity, reinforcing its therapeutic potential in enhancing immune modulation and promoting efficient wound healing [90,113,114,115].

## 5. AMPs in Clinical Development

With some already demonstrating promising results and entering the market [116], several AMPs are currently undergoing clinical trials, reflecting growing interest in their therapeutic potential as alternatives or adjuncts to conventional antibiotics. These trials primarily focus on evaluating the safety, efficacy, and pharmacokinetics of AMPs in treating a range of infections, including skin and soft tissue infections, DFUs, and chronic wound infections [117]. Notable examples include omiganan, a synthetic indolicidin analogue with antimicrobial properties that could benefit patients with atopic dermatitis (AD). A Phase II randomized, double-blind, placebo-controlled trial assessed the pharmacodynamic effects, safety, and clinical efficacy of topical omiganan in patients with mild to moderate AD. Patients were randomized to apply topical omiganan at concentrations of 1% or 2.5%, or a carrier gel, to one target lesion once daily for 28 consecutive days. The findings underscore the therapeutic potential of AMPs as dual-function agents, combining antimicrobial activity with immunomodulatory effects. Compared to the vehicle gel group, both concentrations (1% or 2.5% omiganan) demonstrated the ability to reduce disease severity, alleviate itching, and normalize the skin microbiota. These outcomes reflect its potent antimicrobial properties in restoring microbial balance in dysbiotic skin, a common feature in AD [118].

Moreover, the observed improvements in clinical symptoms, despite the lack of significant changes in inflammatory biomarkers, suggest that AMPs such as omiganan may exert subtle immunomodulatory effects by altering the cutaneous microenvironment and possibly modulating local immune responses [118]. This aligns with emerging evidence that AMPs not only target pathogens directly but also influence host immunity, shaping immune cell behavior, reducing inflammation, and promoting tissue homeostasis [109]. Therefore, the clinical outcomes of omiganan support the translational relevance of AMPs as promising candidates for the treatment of inflammatory skin diseases, offering a multifaceted approach that integrates antimicrobial defense with immune modulation. However, further investigations are necessary to establish more robust clinical evidence regarding the antimicrobial and immunomodulatory effects of omiganan [118].

On the other hand, several peptide-based antibiotics have successfully reached the market and are commercially available, including bacitracin, dalbavancin, vancomycin, and polymyxin E [98,116,119,120]. As shown in Table 2, these FDA-approved peptide antimicrobials demonstrate the clinical viability of peptides in combating bacterial infections. While current research largely emphasizes the structural characteristics and mechanisms of action of AMPs, comprehensive analyses of their clinical translation pathways remain limited. Therefore, it is crucial for future investigations to extend these findings through clinical studies and to establish foundational frameworks that support the successful translation of AMPs into clinical applications.

## 6. Future Prospects and Challenges in Antimicrobial Peptide Research

AMPs present a compelling alternative to conventional antibiotics, combining potent antimicrobial activity with the ability to modulate the immune system. However, despite their potential, AMP-based therapies face significant limitations that hinder clinical translation. These challenges include poor metabolic stability, rapid degradation, toxicity concerns, high production costs, and potential bacterial resistance. Additionally, delivery challenges and regulatory hurdles further complicate their development into viable therapeutic agents [123,124]. Issues regarding stability and toxicity represent the primary obstacles to the implementation of AMPs. Given that AMPs are peptides, their efficacy may be significantly reduced in biological environments due to proteolytic degradation [125]. Furthermore, toxicity concerns arise upon high quantities or poorly designed sequences that may cause unfavorable interactions with the host cell membranes. Consequently, AMP sequencing requires optimization for stability and specificity. This can be achieved through strategies such as cyclization, D-amino acid substitution, or the application of peptidomimetics, which operate analogously to AMPs but are less harmful and more stable [126,127,128]. Further, AMPs face scalability and affordability issues for widespread clinical use, primarily due to their high production costs, particularly for large-scale synthesis or purification. Additionally, the need for specific delivery systems and potential toxicity concerns add to the overall expenses. Synthesizing AMPs can be significantly more expensive than producing conventional antibiotics [129]. The estimated production cost of AMPs at a commercial scale typically ranges between US$50 and US$400 per gram, depending on the specific synthesis method utilized. In comparison, traditional antibiotics such as penicillin G are significantly more cost-effective, with production costs approximating US$10 per kilogram using well-established fermentation processes [31]. Moreover, AMP production using traditional methods such as solid-phase peptide synthesis (SPPS) is often associated with high costs. Similarly, recombinant expression systems, while offering scalability, can also be expensive, with production costs reportedly reaching up to €250,000 (approximately US $270,000, based on the June 2025 exchange rate) per gram. However, efforts are underway to explore cost-effective production methods such as recombinant DNA technology and microbial fermentation [129].

Despite their promising therapeutic potential, the clinical translation of AMPs is impeded by significant regulatory and ethical challenges. Unlike conventional antibiotics, AMPs often reside in a regulatory gray area due to their dual functionality as both antimicrobial agents and immunomodulators, complicating their classification and approval pathways. Regulatory authorities such as the U.S. Food and Drug Administration (FDA) and the European Medicines Agency (EMA) require comprehensive preclinical and clinical data to demonstrate safety, efficacy, pharmacokinetics, and manufacturing consistency. However, the structural complexity and diverse biological activities of AMPs pose challenges in standardizing production, ensuring batch-to-batch quality control, and predicting long-term effects. The clinical utilization of AMPs presents numerous ethical dilemmas, especially concerning the acquisition of informed consent, the criteria for patient selection in clinical trials, and the potential implications of introducing powerful antimicrobial agents into ecosystems already destabilized by extensive antibiotic usage. Additionally, concerns about toxicity, immunogenicity, and stability require rigorous and ethically conducted safety evaluations [117]. Further, the ecological impact of AMP development must be considered, particularly when peptides are sourced from natural environments, to ensure that harvesting or synthesis methods do not disrupt ecosystem balance or biodiversity [130]. To address the aforementioned barriers, there is a critical need for the development of specific regulatory frameworks tailored to peptide-based therapies, adherence to high ethical standards in clinical research, transparent patient communication, and the establishment of international guidelines to support the safe and equitable integration of AMPs into clinical practice [117,131].

The key issue exacerbating these limitations is the lack of recent research progress in addressing these challenges. While AMPs have been extensively studied in preclinical settings, their clinical advancements remain limited, partly due to insufficient funding, regulatory barriers, and the complexity of large-scale manufacturing. The scarcity of new findings on optimizing AMP stability, minimizing cytotoxicity, and improving delivery mechanisms has slowed their integration into mainstream medicine. Moreover, research gaps in understanding AMP interactions with host immunity, microbiome balance, and long-term effects further delay their clinical application. To overcome these barriers, a renewed focus on AMP-based tissue engineering and regenerative medicine (TERM), nanotechnology-based delivery systems, and combination therapies is essential [132]. Additionally, increased investment in clinical trials and regulatory support is crucial for translating promising AMP research into effective, scalable treatments. Without continuous innovation and updated research findings, AMP-based therapies risk stagnation, leaving a critical gap in the fight against antimicrobial resistance and chronic wound infections [124,133,134,135].

Despite significant technological advancements in the biomedical field, the limited availability of advanced computational tools for designing therapeutic AMPs presents numerous challenges that remain to be addressed. Machine learning (ML) has emerged as a promising approach in the development of AMPs. Recent advancements in this field have been documented in recent studies [124,136,137,138,139,140,141]. ‘Smart’ hydrogel, an AI-driven peptide design, unveils a groundbreaking advancement in the application of AMPs for wound healing therapy. Smart hydrogels are intelligent, responsive biomaterials that can adapt to environmental changes, such as pH, temperature, and enzymatic activity, ensuring controlled and sustained AMP release at the wound site. When combined with artificial intelligence (AI)-designed AMPs, these hydrogels can be optimized for enhanced stability, bioavailability, and targeted immune modulation, addressing key limitations associated with conventional peptide therapies. AI-driven algorithms enable precise AMP screening, modification, and optimization, improving their antimicrobial and immunomodulatory functions while reducing toxicity. The combination of machine learning, computational modeling, and high-throughput screening accelerates the discovery of novel AMPs with enhanced therapeutic properties. When embedded in smart hydrogels, these peptides can promote wound healing by reducing infection, modulating inflammation, stimulating angiogenesis, and facilitating tissue regeneration [142,143,144,145,146,147].

This innovative approach paves the way for next-generation wound care solutions, offering personalized and adaptive treatments for chronic wounds. As AI-driven peptide design continues to evolve, the integrations of bioengineering, nanotechnology, and regenerative medicine will further enhance the clinical translation of AMPs, positioning them as key players in the future of wound healing therapies. Additionally, the development of personalized or targeted AMP therapies presents an encouraging prospect in precision medicine, particularly by utilizing their immunomodulatory properties to address patient-specific needs. Instead of a one-size-fits-all approach, this strategy aims to tailor AMP treatments based on an individual’s immune response, infection type, and underlying health conditions. By integrating advanced technologies such as genomics, proteomics, and artificial intelligence, personalized AMP therapies can optimize efficacy, minimize adverse effects, and enhance patient outcomes. For instance, AMPs can be tailored to modulate immune responses in patients with chronic wounds, providing a more targeted and effective therapeutic approach [124,132,147,148].

## 7. Conclusions

AMPs represent a promising and adaptable strategy in the fight against infections, particularly those caused by multidrug-resistant (MDR) pathogens that are unresponsive to conventional antibiotics. With broad-spectrum activity, distinct mechanisms of action, and the potential for synergy with existing treatments, AMPs are emerging as vital additions to the antimicrobial arsenal. Beyond their antimicrobial properties, AMPs play a crucial role in modulating immune responses, offering a dual-function therapeutic approach that not only eradicates pathogens but also accelerates wound healing and promotes tissue regeneration. The strategic regulation of the immune system through AMP-based therapies opens new avenues for targeted, personalized wound care. However, despite their potential, challenges such as toxicity, stability, resistance development, and large-scale production must be addressed through sustained research efforts. As the global health community confronts the escalating threat of antibiotic resistance, advancing peptide-based immunomodulation through interdisciplinary collaboration and consistent investment will be key to unlocking the full therapeutic promise of AMPs, shaping a future of more effective infection control and improved tissue repair strategies.

## Figures and Tables

**Table 1:** Dual roles of AMPs in modulating immune response and antimicrobial activities in wound healing.

Antimicrobial Peptide	Antimicrobial Mechanisms	Immunomodulatory Function	References
Human β-defensins (hBDs)	Exhibit broad-spectrum antimicrobial activity against various pathogens (i.e., bacteria, fungi, and viruses)	Release pro-inflammatory cytokines and chemokines that attract immune cells such as neutrophils and macrophages to the wound site, modulate macrophage polarization, and suppress neutrophil apoptosis	[83,84,85,86]
Tet213-CN	Improves bacterial phagocytosis	Promotes M1 macrophage polarization, increases intracellular ROS generation and proinflammatory cytokine secretion, and modulates macrophage polarization	[87,88]
Human cathelicidins LL-37	Directly kill bacteria, fungi, and viruses by disrupting their cell membranes	Regulate inflammation by balancing between pro-inflammatory and anti-inflammatory actions, modulate macrophage polarization, act as a chemoattractant to guide immune cells such as neutrophils to the wound site, stimulate cytokine production, bridge innate immunity and adaptive immunity, and suppress neutrophil apoptosis	[86,89,90]
Mast-MO	Compromises bacterial cell membrane integrity	Stimulates cytokine production, reduces excessive inflammation promotes a balanced immune response by suppressing pro-inflammatory factors (e.g., TNF-α and IL-6)	[91]
Andersonin-W1 (AW1)	Directly kills pathogens and prevents biofilm formation	Balances inflammation, enhances immune cell recruitment, promotes tissue repair, and modulates inflammation by macrophages via the TLR4/NF-κB molecular axis by directly binding to TLR4 in the macrophage extracellular region	[92]
Antimicrobial peptide derived from insulin-like growth factor-binding protein 5 (AMP-IBP5)	Exhibits broad-spectrum antimicrobial effects against Gram-positive and Gram-negative bacteria, kills antibiotic-resistant strains such as MRSA and *Pseudomonas aeruginosa*, and inhibits biofilm formation	Regulates inflammation and induces macrophage polarization	[93,94]
Human α-defensins	Directly kill or inhibit bacterial growth via membrane disruption and inhibition of bacterial cell wall synthesis	Act as chemokines to recruit cells such as neutrophils, eosinophils, mast cells, monocytes, and lymphocytes to the infection or wound site and suppress neutrophil apoptosis	[86,95]
Thrombocidin-1-derived peptide TC19	Eradicates superficial multidrug-resistant bacterial strains, including *Staphylococcus aureus* and *Acinetobacter baumannii*	Induces neutrophil chemotaxis	[96]

**Table 2:** Examples of FDA-approved peptide-based antimicrobial medications that are available on the market.

Peptide-Based Antimicrobial Medication	Brand Name	Application	Target Species	References
Bacitracin	Neosporin	To treat skin infections	Mainly Gram-positive bacteria, including *Staphylococcus*, *Streptococcus*, *Corynebacterium*, *Clostridium*, and *Actinomyces*	[98]
Vancomycin	Firvanq, Vancocin	To treat serious Gram-positive infections, including MRSA infections	*Staphylococcus aureus*	[116]
Dalbavancin	Dalvance, Xydalba	To treat skin infections	Gram-positive pathogens, including *Staphylococcus aureus* (including MRSA), *Streptococcus pyogenes*, *Streptococcus agalactiae*, and *Enterococcus faecalis* (vancomycin-susceptible strains)	[116,119]
Polymyxin E	Colistin	To treat multidrug-resistant (MDR) Gram-negative bacterial infections	*Pseudomonas aeruginosa*, *Acinetobacter baumannii*, and *Klebsiella pneumoniae*	[116,120]
Bulevirtide	Hepcludex	To treat chronic Hepatitis D (HDV) infection in adults with compensated liver disease	Hepatitis D virus	[116,121]
Enfuvirtide	Fuzeon	An HIV-1 fusion inhibitor used in the treatment of human immunodeficiency virus (HIV) infection, primarily prescribed for patients who have developed resistance to other antiretroviral therapies (ART)	Human immunodeficiency virus (HIV)	[116,122]

## Data Availability

Not applicable.
